# Fundamental edge broadening effects during focused electron beam induced nanosynthesis

**DOI:** 10.3762/bjnano.6.47

**Published:** 2015-02-16

**Authors:** Roland Schmied, Jason D Fowlkes, Robert Winkler, Phillip D Rack, Harald Plank

**Affiliations:** 1 Graz Centre for Electron Microscopy, 8010 Graz, Austria; 2 Center for Nanophase Materials Sciences, Oak Ridge National Laboratory, Oak Ridge, Tennessee 37831, USA; 3 Department of Materials Science and Engineering, University of Tennessee, Knoxville, Tennessee 37996, USA; 4 Institute for Electron Microscopy and Nanoanalysis, Graz University of Technology, 8010 Graz, Austria

**Keywords:** focused electron beam induced deposition, nanofabrication, platinum, simulation

## Abstract

The present study explores lateral broadening effects of 3D structures fabricated through focused electron beam induced deposition using MeCpPt(IV)Me_3_ precursor. In particular, the scaling behavior of proximity effects as a function of the primary electron energy and the deposit height is investigated through experiments and validated through simulations. Correlated Kelvin force microscopy and conductive atomic force microscopy measurements identified conductive and non-conductive proximity regions. It was determined that the highest primary electron energies enable the highest edge sharpness while lower energies contain a complex convolution of broadening effects. Moreover, it is demonstrated that intermediate energies lead to even more complex proximity effects that significantly reduce lateral edge sharpness and thus should be avoided if desiring high lateral resolution.

## Introduction

Focused electron beam induced deposition (FEBID) has attracted increasing attention due to capability to directly write functional (3D) structures with nanometer resolution [1–3]. Compared to lithography-based methods, FEBID does not require pre- or post-growth treatments and can be used on non-flat surfaces. This makes this technique a potential candidate for an enabling nanofabrication technology. The technique relies on the local nano-synthesis of precursor molecules by a focused electron beam and its subsequent electron emission from the substrate and the deposit itself [1,3–6]. Typically, a gaseous precursor is brought into the chamber via a dedicated gas injection system where it adsorbs at the surface, undergoes random diffusion and desorbs again after a system-dependent residence time [1,3–4,7–19]. A major problem of FEBID, however, is the large amount of carbon impurities that often stem from incompletely dissociated precursor molecules or non-volatile fragments [10]. As these contents can reduce or even mask the intended functionality [4,6] strong efforts have been made to optimize the purification processes [6,20–33]. Recently, Geier et al. demonstrated an approach that finally allows a full purification at high rates without elevated temperatures and/or highly reactive gases after initial fabrication [32]. While this approach is ideal for small structures, Sachser et al. demonstrated a catalytic purification approach without the need of a scanning e-beam that is, hence, highly suited for the purification of large areas [33]. Despite these purity issues, a variety of applications have been demonstrated for different precursors such as nano-optics [34–35], magnetic storage, sensing and logic applications [36–40], and nanoscale stress/strain or gas sensors [28,41–42]. While highly accurate and reproducible surface morphologies are essential for some of these applications, e.g., nano-optics, all of these applications require predictable and reproducible shape control in the context of high integration densities due to the ongoing downscaling trends. While Hari et al. recently demonstrated the fabrication of sub-20 nm structures by a careful experimental setup [43], Arnold et al. described the role of backscattered electrons (BSEs) generated by the growing deposit itself and its consequences on lateral broadening effects [44]. Both studies, however, used highly defined quasi-1D or quasi-2D structures as ideal test models and/or with the aim of unique lithography alternatives. For many applications, however, 3D deposits are required and thus more complex proximity effects emerge due to the extensive electron trajectories, which have been basically demonstrated in experiments and simulations [18]. In order to push this technique further towards the intrinsic limits, a detailed knowledge of broadening effects, their origins and the scaling behavior for edge-effects of 3D structures needs to be acquired.

In this study, we focus on such broadening effects for 1 × 1 µm deposits with heights below 140 nm. We analyze the morphology, the chemistry and the electric properties of the proximity effects by a multi-technique approach and complement the experimental data with Monte Carlo electron–solid simulations. Furthermore, we focus on the qualitative scaling behavior of proximity effects as a function of primary electron energy and deposit height or thicknesses. Beside the discussion of fundamental broadening effects, we derive ideal and non-ideal parameter ranges from the scaling behavior as a practical output of this study.

## Experimental

FEBID was performed with a FEI Nova200™ dual beam microscope (DBM) equipped with a FEI gas-injection-system (GIS) for Pt deposition, arranged at an angle of 52° and a vertical distance of 120 µm to the substrate with an exact alignment of the GIS main axis with respect to the deposition area as described in detail by Winkler et al. [45]. For the deposition of PtC (MeCpPt(IV)Me_3_) precursor was used at a reservoir temperature of 45 °C (heated for at least 45 min prior to any experiment). Two types of substrates have been used: 1) bare B-doped Si substrates with a 500 nm SiO_2_ top-layer providing a root mean square (RMS) surface roughness values of less than 0.1 nm, and 2) the same substrates with 60 nm Au electrodes, fabricated through electron-beam lithography using a 3 nm Cr interfacial adhesion layer. While the former were used for atomic force microscopy (AFM) and Kelvin force microscopy (KFM) investigations, the latter have been used for conductive-AFM (C-AFM) measurements. All substrates were taken from sealed wafer boxes and were immediately transferred to the dual beam microscope followed by overnight pumping towards a target chamber pressure of (2–3)·10^−6^ mbar. Prior to patterning, the GIS was opened for about 2 min to establish stable precursor conditions at the surface at a chamber pressure of (1–2)·10^−5^ mbar. All structures have been fabricated by using stream-files that were generated through a patterning algorithm custom-written in C++ [46]. Unless otherwise noted, 1 × 1 µm^2^ squares with variable heights have been used for investigations. Primary electron energies and beam currents were 5 keV/98 pA, 10 keV/130 pA, 15 keV/140 pA, 20 keV/150 pA, 25 keV/150 pA and 30 keV/150 pA (in contrast to the finely adjustable beam energies, the beam current can only be chosen in steps due to technical reasons). All patterns used a serpentine scanning strategy, constant pixel dwell times (DTs) of 100 µs and pixel point pitches (PoPs) of 50% beam overlap resulting in distances of 14.9 down to 7.1 nm. Different thicknesses have been realized by varying the number of patterning loops. Beam currents, DTs and PoPs were based on previous studies, suggesting a widely balanced working regime without strong excess of electrons or molecules [10,44–45,47]. Preliminary experiments were performed without precursor gas by using the same patterns at the highest exposure times revealing low carbon contamination in the sub-nanometer range. After successful fabrication the deposits were not e-beam inspected but immediately transferred to the AFM system. AFM and KFM experiments have been performed with a Dimension 3100 System (Bruker Nano), equipped with a Hybrid scan head and operated with a Nanoscope IV controller together with the C-AFM application module for the respective measurements. OMCL-C240-TS and ASYELEC-01 (TiIr surface coating) cantilevers have been used for AFM/KFM (2-pass tapping mode) and C-AFM (contact mode) measurements, respectively. The latter experiments have been carried out at voltages between −12 and +12 V depending on the individual purpose. All AFM-based experiments have been performed in a glove box under inert nitrogen atmosphere, which reduces the H_2_O wetting layer on the surface, particularly beneficial for high-resolution KFM measurements.

Monte Carlo simulations for radial BSE distributions have been performed by using the software package CASINO v2.42 [48] assuming the AFM-measured deposit values and a typical PtC_5_ chemistry for such FEBID deposition conditions. All emission profiles were subsequently re-normalized to areal values to access the real radial distributions.

## Results

### Morphology

**Figure 1:**
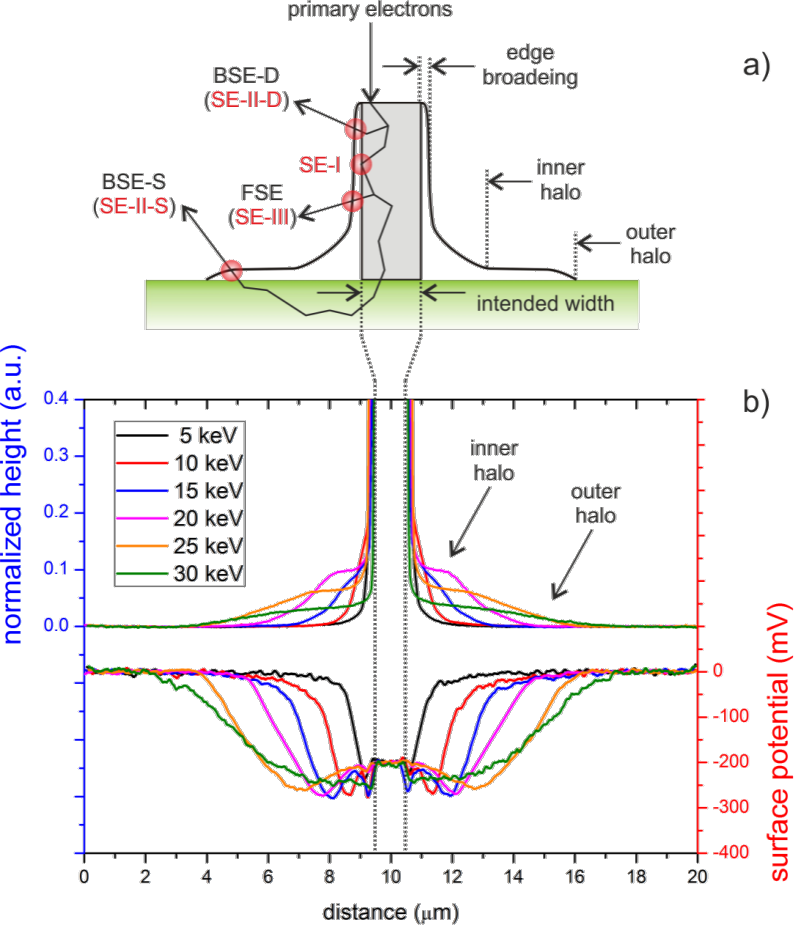
(a) Classification of proximal shapes (right hand side). The grey box indicates the intended deposit while the black curve schematically summarizes the proximal effects. The left hand side indicates the different electron species depending on their exit position. The notation S and D stands for substrate- and deposit-related species, respectively, which holds for BSE and related SE-II contributions. Forward scattered electron (FSE)-related SEs are denoted as SE-III. (b) Normalized AFM height cross sections of similar thickness deposits (65 ± 6 nm) for different primary energies (upper graphs, left Y axis) together with KFM-based surface potential data offset to 0 V for the values of SiO_2_ (lower graphs, right Y axis).

**Figure 2:**
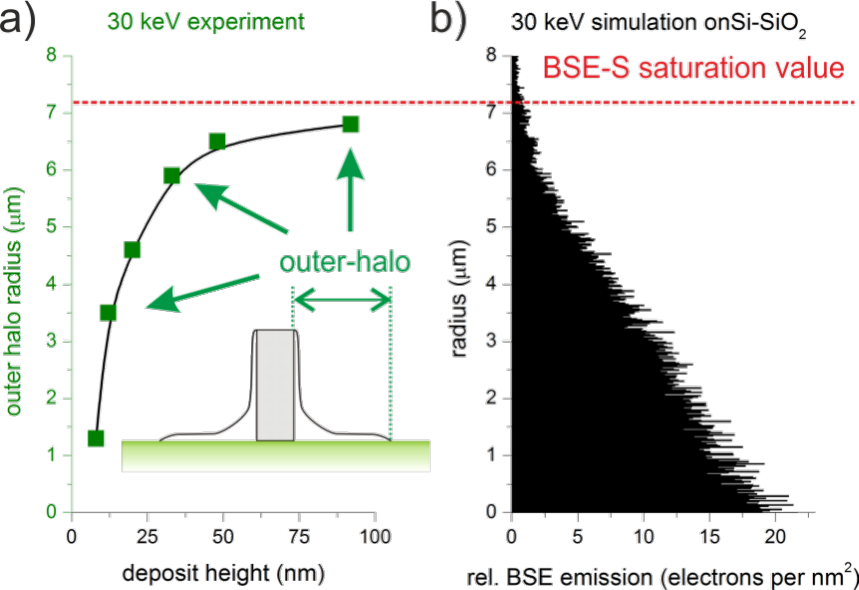
(a) Radius of the outer halo (AFM-based) of 30 keV PtC deposits as a function of the central pad thickness. (b) Simulated BSE-S radius for 30 keV electrons in Si–SiO_2_.

At first, a full set of FEBID structures have been deposited with different primary electron energies (5–30 keV), similar beam currents (98–150 pA) and varying deposit thicknesses between 5 and 132 nm. The upper part of Figure 1b (left Y axis) shows a comparison of normalized AFM height cross sections for similarly thick deposits (65 ± 6 nm) to reveal the energy dependent evolution of proximity deposition. Although different in their dimensions the proximal shapes can be classified by three distinct features as schematically shown on the right hand side in Figure 1a: 1) small edge-broadening (EB), which is always found in the range of 20–60 nm per side in agreement with single line broadening effects recently described by Arnold et al. [44]; 2) a large outer halo (OH), which is extremely thin (less than 5% of the deposit height, see Figure S1, Supporting Information File 1); and 3) an inner halo (IH), which changes its shape depending on the primary energy. As can be seen, the inner halo reveals a distinct plateau for intermediate electron energies around 20 keV, which strongly reduces the achievable lateral deposit edge sharpness (magenta line in Figure 1b). For decreasing primary energies the plateau gets higher but more narrow (e.g., blue line in Figure 1b for 15 keV). In contrast, higher primary energies lead to flatter plateaus with increasing radius finally resulting in a broadening that is dominated by the outer halo (green line in Figure 1b for 30 keV). Figure 2 shows the thickness-dependent radius of the outer halo (AFM-based) for 30 keV deposits (a) together with the simulated backscattered electron (BSE) radius (b) derived from the used Si–SiO_2_ substrate (simulated by the Monte Carlo package CASINO [48]). As evident in Figure 2, the experimentally measured outer halo radius approaches the simulated value (indicated by the dashed red line), which reveals substrate-related BSEs (BSE-S) to be involved in the formation of the outer halo. Considering the cross-section of MeCpPt(IV)Me_3_ precursor molecules [3,17,49–50] with its maximum clearly below 1 keV, it is very likely that secondary electrons type II (SE-II) are mainly responsible for the dissociation although a direct dissociation through BSE-S is also likely to contribute. Hence, it can be concluded that the outer-halo can predominantly be assigned to substrate-related BSE-S and SE-II, further denoted as SE-II-S (see also the left hand scheme in Figure 1a). A detailed discussion for the edge broadening, EB, and in particular for the inner halos, IH, is given later as more experimental data and simulations are needed for a comprehensive explanation.

### Functionality

In the following a two-step approach is followed: First, the surface potential, which reflects the chemical composition and its electronic properties, and, subsequently, the electric conductivity are mapped. The combination of both measurements allows one then to derive the scaling behavior of functional (electrically conductive) and non-functional (electrically insulating) proximity regions in dependence on the primary electron energy and the deposit thickness.

**Figure 3:**
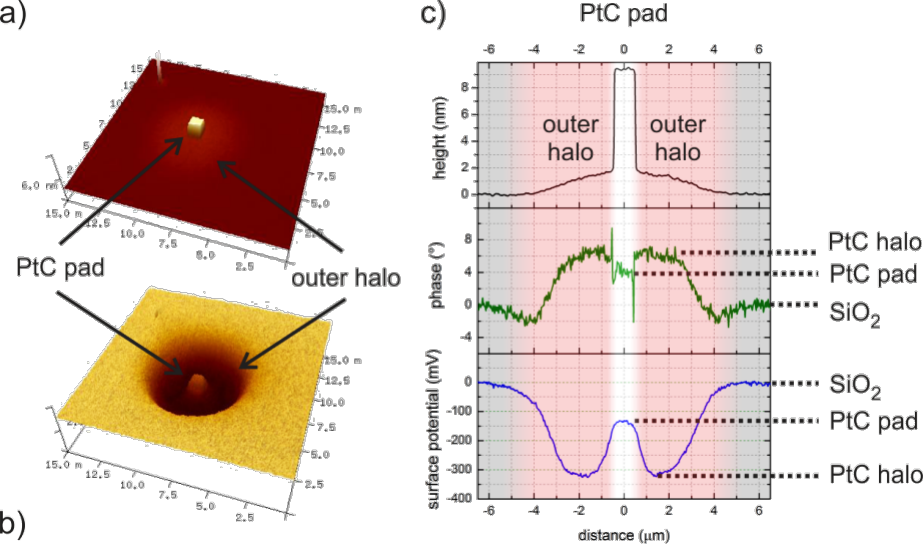
Representative AFM height image (a) of a 9 nm thick PtC deposit on Si–SiO_2_ fabricated at 25 keV together with the correlated surface potential distribution (b) via KFM. (c) Corresponding cross sections for height (top), tapping phase (center) and surface potential (bottom). The colors illustrate the intended PtC pad (white), the outer halo (red) and the SiO_2_ substrate (grey).

To access the chemical properties of the deposits, Kelvin force microscopy (KFM) was conducted as it provides a laterally resolved measurement of the variations in surface potential. Figure 3 gives a representative AFM height image (a) together with the corresponding surface potential (b) of a ca. 9 nm thick deposit fabricated at 25 keV. Correlated cross sections are shown in Figure 3c for the height (top), the tapping phase (center, green), and the surface potential (bottom, blue). As can be seen the surface potential reveals three different levels: 1) the SiO_2_ substrate (offset to zero); 2) the deposit with a potential difference of about −150 mV; and 3) another level for the outer halo at −300 mV.

**Figure 4:**
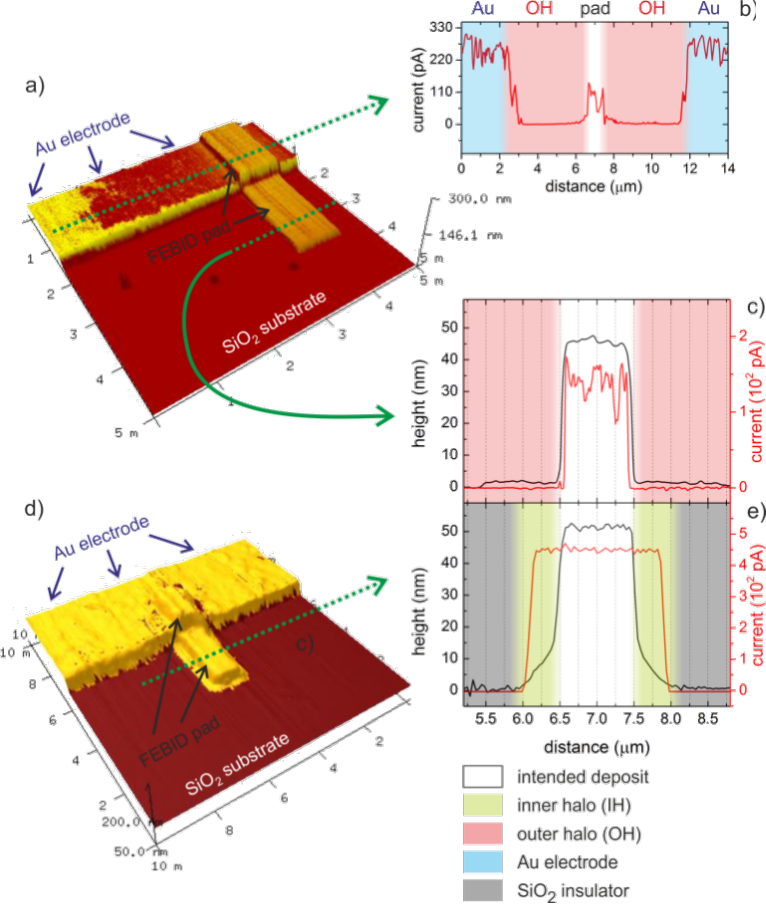
AFM height images with overlaid current information of PtC pads deposited on an conductive Au electrode extending to the insulating SiO_2_ substrate for a 30 keV (a) and 5 keV (d) deposit. Current cross sections for the 30 keV deposit (a) are given in (b) and (c) for Au and SiO_2_ areas, respectively, revealing the nanometer thick outer halo OH as insulating. In contrast, (e) shows correlated height (black, left axis) and current (red, right axis) cross sections for the 5 keV deposit (d) revealing the proximity deposition as fully conductive. The individual shadings in the cross sections are specified bottom right.

The essential information is the fact that the surface potential of the outer halo level is *below* those of both the PtC pad and the SiO_2_. This excludes the idea that the formation of this intermediate level is a simple convolution of substrate and central deposit. Once the outer halo gets thinner at increasing radii, the associated surface potential gets increasingly dominated by the SiO_2_ substrate underneath and finally approaches the same value. As KFM gives insight into the electronic band structure, the distinct potential of the outer halo indicates different chemistries and/or functional properties compared to the central PtC deposit. This is further supported by the tapping phase signal (central plot in Figure 3c) that suggests different mechanical properties for the outer halo. As KFM is not able to reveal the electric conductivity, conductive-AFM (C-AFM) measurements were carried out. For that, FEBID structures were deposited across the edge between a grounded Au electrode and the highly insulating SiO_2_ substrate as shown in Figure 4a for a 40 nm thick 30 keV deposit. The color scale corresponds to the correlated current signals through sample/deposit/AFM tip and brighter colors indicate higher conductivities. Figure 4b gives a current cross section along the upper dotted green line in Figure 4a revealing three different levels: 1) highest currents for the conductive Au electrode as expected (blue region); 2) reduced currents at the central deposit area due to the lower conductivity of as-deposited PtC structures (white regions) [28,31,41]; and 3) zero current in between according to the darkest areas in Figure 4a (red zones). The latter can be assigned to the very thin outer halo (less than 2 nm thickness), which corresponds well with the observation of different surface potentials for the same regions. To confirm the finding of a non-conductive outer halo, another current cross section on the insulating SiO_2_ substrate is shown in Figure 4c (along the lower dotted line in Figure 4a) also revealing electrical conductivity only for the central deposit (white area). From this data we can conclude that lower surface potentials (KFM) indicate lower electric conductivities (C-AFM), further denoted as “functionality”.

**Figure 5:**
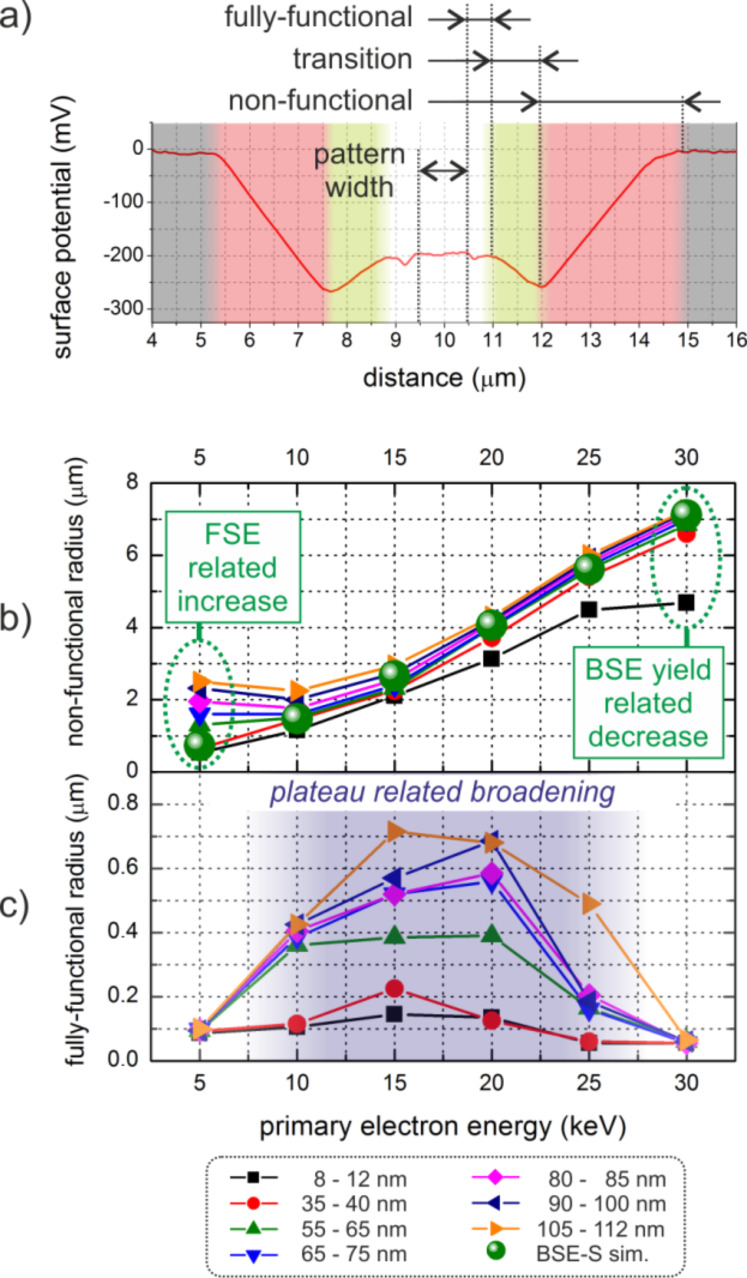
(a) Functional classification of proximity deposition based on KFM measurements. (b) and (c) show the energy- and thickness-dependent radii of the non-functional and the fully-functional radius, respectively. A detailed explanation is given in the main text.

Following this correlation approach we can classify the lateral functionalities based on KFM data as summarized in Figure 5a: 1) a fully-functional range for potential values very similar to the central deposit (white area); 2) a non-functional radius starting from lowest potential values to highest detectable deviation from the substrates (red zone); and 3) a transition area between (1) and (2) (green range). Figure 5b summarizes the non-functional radii as a function of the primary energy for different thicknesses (see bottom legend). Associated simulations of substrate-related BSE-S radii (green spheres) are in good agreement with the experimentally observed radii of the outer halo, which further confirms their strong correlation. The decaying behavior for thinnest pads at highest energies (encircled on the right hand side) can be explained by the decreasing number of primary electrons together with the low BSE yield at such high primary energies. This leads to very low areal BSE emission at the highest radial positions which can (partly) dissociate precursor molecules, however, below the KFM detection sensitivity. On the other hand, the increasing non-functional radius for thicker deposits at lower energies (encircled on the left hand side) is a result of increasing FSE contributions as will be discussed in the following section.

Interestingly, the fully-functional radius (Figure 5c) shows the smallest values at high and low primary energies while considerable broadening is observed for intermediate energies. Correlation with the morphological data reveals the distinct proximity plateaus (see Figure 1b and supplement Figure S2) as partly electrically functional areas as indicated by the blue shading in Figure 5c. The origin of the plateaus is discussed in the following section.

## Discussion

At this point it is important to note that the present study primarily focuses on the qualitative description of energy- and height-dependent proximity effects with respect to morphology and functionality. The latter describes the electrical conductivity and is derived from combined C-AFM and KFM measurements. The absolute values for halo heights and functional radii might slightly change for different precursor regime conditions but show the same qualitative scaling behavior. The discussion starts with highest primary energies due to simplicity further expanded by the more complex situation for low primary energies. Finally, intermediate energies are discussed, which show distinct morphological proximity plateaus as evident in Figure 1.

### High primary energies

**Figure 6:**
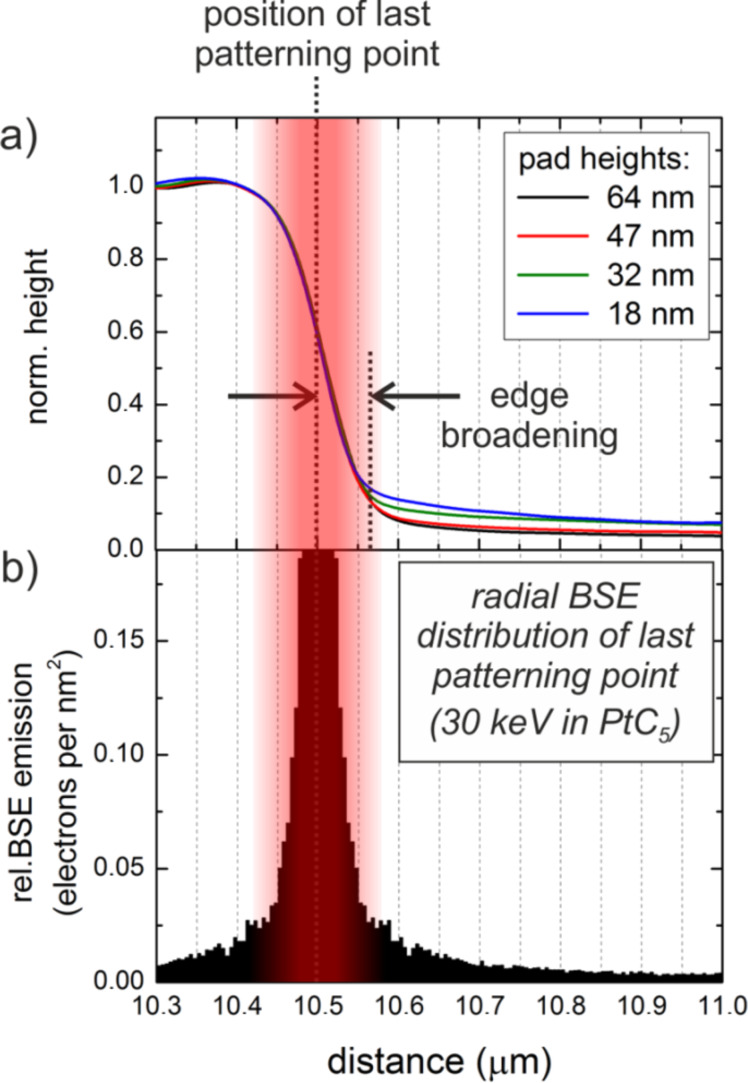
Edge-broadening effect for 30 keV deposits of different thickness. (a) shows a normalized height representation with the last patterning point at 10.5 µm indicated by the vertical dotted line. (b) shows the simulated radial BSE emission in PtC_5_ (BSE-D) for the last patterning point revealing the symmetric edge broadening in agreement with the respective BSE-D distribution (red shades).

First, we focus on the non-functional outer halo (C-AFM, Figure 4) revealing distinctly different surface potentials compared to the central deposit (KFM, Figure 3). It is well-known that the dissociation of the MeCpPt(IV)Me_3_ precursor molecules is not a single step process [2–3,16–17]. Thus, we suggest the low areal flux of substrate related BSE-S/SE-II-S at high primary energies results in a partial dissociation, e.g., single methyl dissociation [51], which hinders the formation of Pt nanograins. This is further supported by the very low thickness of the OH [44]. This can explain both, the distinctly different surface potential as well as the insulating characteristic of the outer halo observed via KFM and C-AFM, respectively. To understand the edge-broadening effect a closer look at the edge morphology is required as shown in Figure 6a, which is a normalized plot of 30 keV deposits with different thicknesses. The dotted vertical line gives the last patterning point at the edge (intended patterning edge) revealing the final slope to be symmetrically distributed at half maximum. Previous studies by Arnold et al. [44] revealed that the achievable width of single lines is determined by BSE/SE-II contributions from the deposit itself (BSE-D/SE-II-D). Analogous to that work, Figure 6b shows the simulated radial BSE-D distribution for the outermost patterning point (30 keV in 30 nm PtC_5_ on Si–SiO_2_ (500 nm)). The direct comparison (highlighted by the red shading) shows good agreement between the lateral BSE-D spread and the symmetric slope determined via simulations and experiments, respectively. This strongly suggests that the edge-broadening (EB) outside of the intended deposit is influenced by SE-II contributions, which are triggered by BSE originating from the deposit. Detailed KFM analyses of EB areas reveal the same surface potential as the central deposit suggesting a fully-functional character (same electric properties as the central deposit).

The observation of an increasing slope inside the intended footprint is described in detail by Winkler et al. [52] and can be mainly attributed to the patterning itself: While volume growth rates at the very central patterning points are supported by deposit related BSE/SE-II contributions in X and Y, patterning points at the edges and corners show a reduced number of neighboring patterning points [18]. The resulting decrease of deposit-related BSE/SE-II contributions then leads to decreased net volume growth rates at the outermost patterning edges and corners [18,52].

In summary, the observed edge-broadening for high primary electron energy is an unavoidable effect ultimately contributing to the finally achievable, fully-functional edge sharpness. It is important to mention that absolute values of the proximity radii strongly depend on the experimental setup as impressively demonstrated by van Oven et al. and Hari et al. [15,43].

### Low primary energies

**Figure 7:**
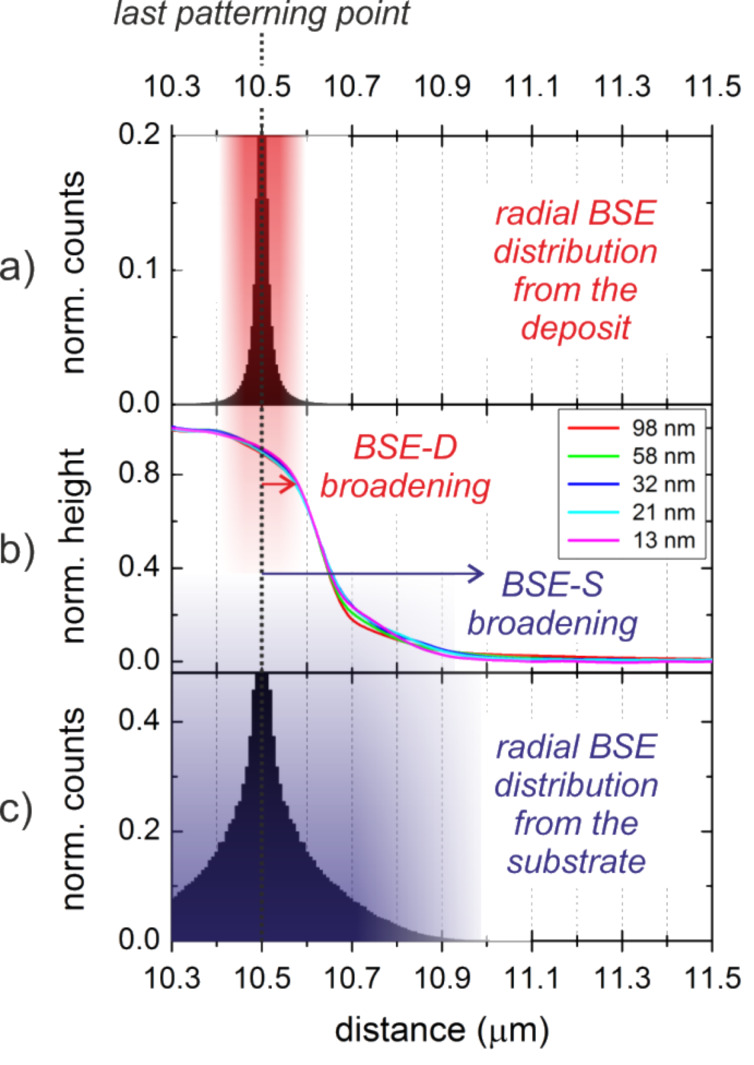
Broadening effects for 5 keV deposits of different thickness. (b) shows the normalized height representation (absolute pad thicknesses are specified in the legend) with the last patterning point at 10.5 µm (dotted vertical line). (a) shows the simulated BSE-D distribution of 5 keV electrons in PtC_5_ for the last patterning point which again is in good agreement with the symmetric edge broadening (red shades). (c) gives the simulated BSE-S distribution of 5 keV electrons in the substrate which can explain the outer proximity deposition (blue shades).

**Figure 8:**
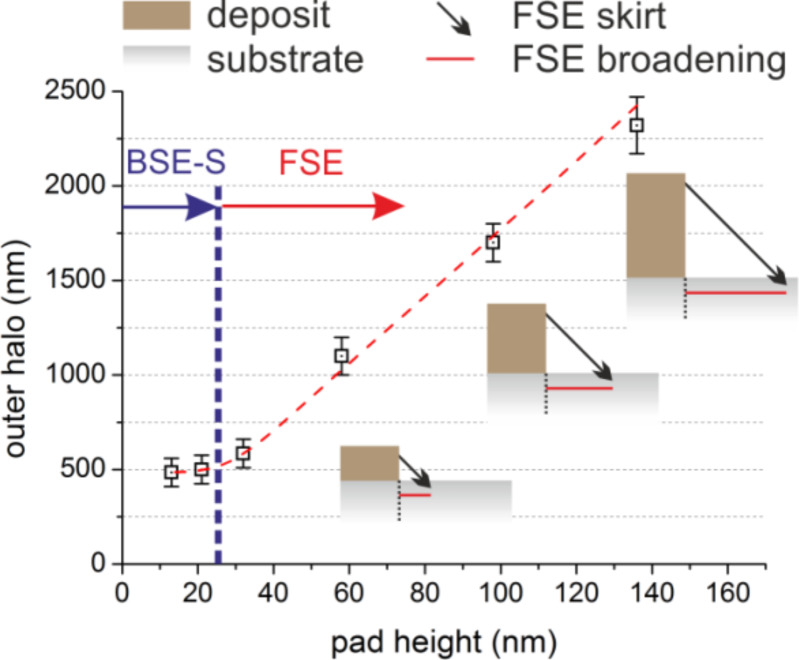
Outer-halo behavior for increasing pad thicknesses of 5 keV deposits (squares) together with an FSE-related scheme to explain the linear increase for thicker deposits (details are given in the main text).

Next, the broadening effects for lowest energies and increasing deposit thicknesses are discussed. Figure 7 shows a normalized set of AFM height cross-sections of 5 keV deposits of different thickness (b) with the last patterning point at the edge being indicated by the dashed line. Evidently, a symmetric slope around the pattern edge can be identified (red shading) followed by a strong decay (blue shading). The symmetric slope can again be explained by the radial BSE distribution originating from the deposit (BSE-D and entailed SE-II-D) as shown for the last patterning point in Figure 7a (correlated red shading). The large scale proximity deposition can clearly be attributed to substrate-related BSE/SE-II electrons (BSE-S/SE-II-S) as shown by correlated simulations in Figure 7c (correlated blue shades). Very careful KFM analyses with respect to largest detectable outer-halo radii revealed a behavior which is shown in Figure 8 by the squares. For deposit thicknesses below 30 nm, outer-halo radii of around 450 nm are found, which are in good agreement with the BSE-S radius shown in Figure 7c (schematically indicated by the vertical dashed line in Figure 8). Thicker deposits, however, led to an almost linear increase of the outer halo (dashed red line). To explain this proximity deposition the small interaction volume for low energy electrons in the growing deposit has to be considered, which leads to forward scattered electrons (FSE) at the sidewalls followed by re-entry in the surrounding areas [18,53]. Please note that similar to the SE-II emission we expect FSE triggered type-III secondary electrons (SE-III) as the predominantly dissociating species. As indicated by the inset schematic in Figure 8, the growing deposits (brown boxes) lead to an almost linearly scaling FSE radius (red lines), which corroborates the experimentally observed scaling of the outer halo. This strong height-dependent broadening also explains why the non-functional radius increases as shown in Figure 5b (encircled green).

In summary, we conclude from this section that for low energy deposits, the highest lateral sharpness can only be achieved for thin deposits for which the FSE contributions are negligible. In such a situation the deposit-related BSE-D/SE-II-D broaden the edges (Figure 7a). This broadening is overlaid by a substrate-related broadening due to BSE-S/SE-II-S contributions (Figure 7c). Thicker deposits lead to increasing FSE/SE-III contributions that further broaden the deposit although the related halo thickness is very thin (less than 2 nm for highest deposits of 136 nm). The non-conductive character is attributed to the low FSE/SE-III re-entry yield and thus to a low dissociation rate. As a general rule deduced here, low primary energies should only be used for very thin deposits in order to achieve sharp deposit edges.

### Intermediate primary energies

**Figure 9:**
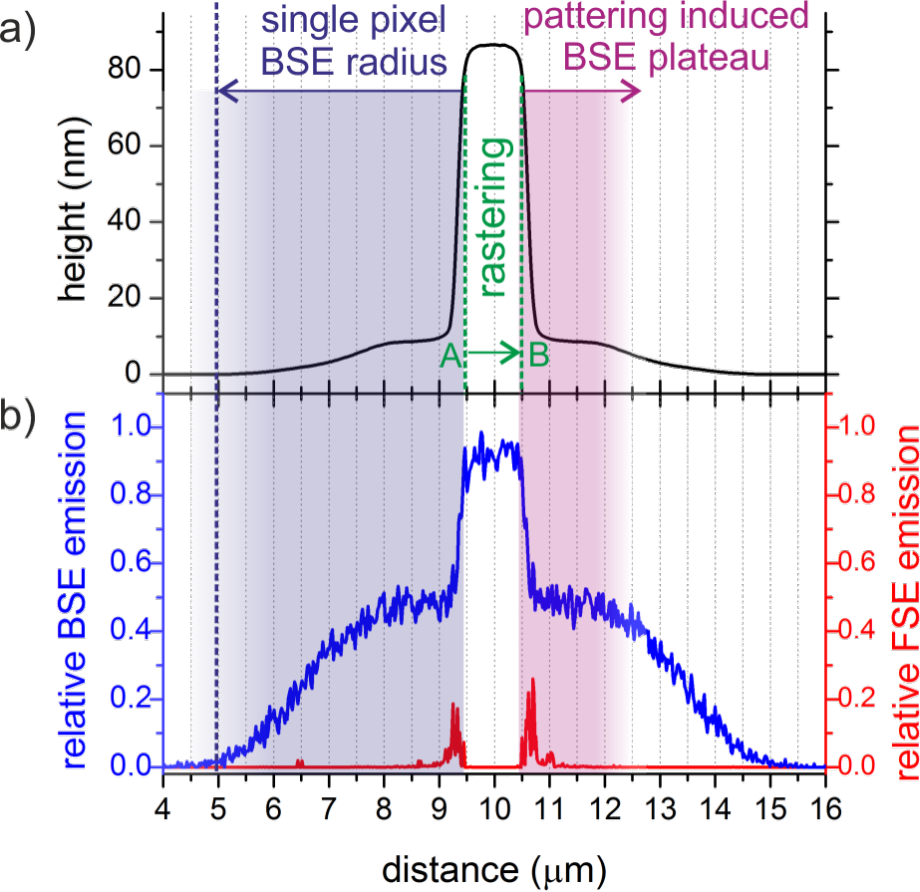
(a) AFM height cross section of a 20 keV deposit. (b) cumulative BSE emission (blue, left axis) and FSE emission and re-entry (red, right axis) contributions after scanning from A to B (indicated in (a)) in normalized plot. While FSE contributions can be neglected (red) due to the larger interaction volume, BSE (blue) and triggered SE-II emission can explain the plateau formation by means of a pattern reflection as discussed in the main text.

Finally, the distinct plateaus for intermediate primary energies (Figure 1b) and the corresponding strongly varying functionalities (Figure 5d) need to be explained. As evident in Figure 9a, 20 keV deposits reveal a distinct morphological plateau outside the intended deposit. To explain this feature, the cumulative BSE and FSE emission during a patterning line from point A to B (indicated in green) has to be considered. This is shown in Figure 9b by a normalized representation in which the FSE simulation takes exiting and re-entering electrons into account as well. As is evident, FSE contributions (red) are irrelevant for the plateau formation due to the low scattering of 20 keV electrons in sub-100 nm PtC deposits. The cumulative BSE distribution (blue), however, shows two different features. As indicated by the blue shading on the left side of the intended deposit, the substrate-related BSE-S radius is in the range of about 5 µm in agreement with the outer-halo radius. The more important features are the plateau-like shapes outside the intended deposit as highlighted by the right hand red shade in Figure 9. These shapes evolve as a consequence of the patterning process itself: When rastering from A to B, the lateral BSE-S distribution from the substrate is a superposition of each single point along the patterning line leading to the blue BSE emission curve. As evident the profile shape is in very good agreement with the experimentally observed plateau (black curve) and can be understood as pattern reflection outside the intended structure (red shade). Once, point B is reached a continuous decay follows with a total radius of about 5 µm in agreement with simulated BSE radius stemming from the substrate (blue shades). Please note, the very distinct plateau formation at 20 keV is a complex interplay between primary electron energy, the substrate material used and the patterning footprint geometry and by that complicated to predict. As general rule, however, it can be deduced that intermediate primary electron energies should be avoided when aiming for highest lateral resolution (see also Figure 5c).

## Conclusion

In conclusion, we have qualitatively studied side-wall broadening effects for three-dimensional FEBID deposits using MeCpPt(IV)Me_3_ precursor on Si–SiO_2_ substrates. It is found that highest primary electron energies lead to sharpest deposit edges widely independent of the thickness in a sub-140 nm regime. If samples are not compatible with high energies, low primary energies are an alternative, however, with a strong thickness dependency due to increasing contributions from forward scattered electrons (see Figure S3, Supporting Information File 1). The important finding of this study, however, is the fact that intermediate primary energies can lead to significant edge broadening as a consequence of substrate-related backscattered electrons resulting in distinct plateaus around the intended deposit. Finally, as previously observed for quasi-1D single lines [44], an unavoidable edge broadening occurs as the result of deposit-related backscattered electrons. It is important to note that absolute values for the observed broadening effects strongly depend on the deposit chemistry (different precursors), the working regime (beam current, dwell times, point pitches) as well as on the substrate type as spatial electron trajectories change with these properties. Nevertheless, the findings of this study give a general qualitative insight into the behavior and scaling of proximity deposition that ultimately limits the achievable edge sharpness and by that lateral resolution (see Figure S4, Supporting Information File 1).

## Supporting Information

File 1 Additional experimental data.
