# Supplementary material for: Fundamental edge broadening effects during focused electron beam induced nanosynthesis
Source: Beilstein J Nanotechnol. 2015 Feb 16;6:462–71. doi: 10.3762/bjnano.6.47 (PMC4362041; doi:10.3762/bjnano.6.47)
Supplement: File 1 — Additional experimental data. [file Beilstein_J_Nanotechnol-06-462-s001.pdf]

Supporting Information

for

Fundamental edge broadening effects during focused electron beam induced nanosynthesis

Roland Schmied[1], Jason D. Fowlkes[2,3], Robert Winkler[1], Phillip D. Rack[2,3] and Harald Plank*,§[1,4]

Address: [1]Graz Centre for Electron Microscopy, 8010 Graz, Austria, [2]Center for Nanophase Materials Sciences, Oak Ridge National Laboratory, Oak Ridge, Tennessee 37831, USA, [3]Department of Materials Science and Engineering, University of Tennessee, Knoxville, Tennessee 37996, USA and [4]Institute for Electron Microscopy and Nanoanalysis, Graz University of Technology, 8010 Graz, Austria

Email: Harald Plank* - harald.plank@felmi-zfe.at

* Corresponding author

§Tel: +43 316 873 8821

Additional experimental data

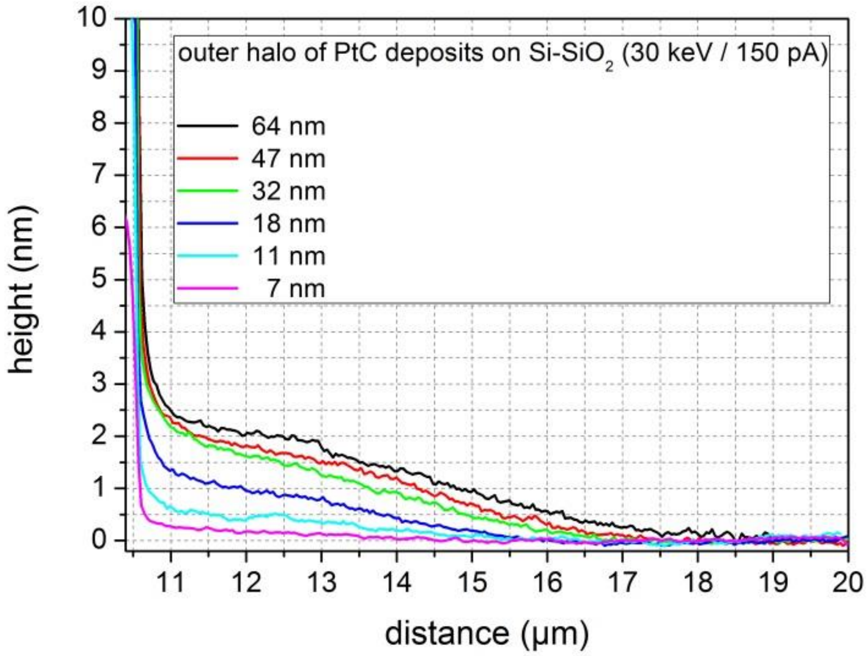

Figure S1: Detailed view of proximal layer shape and thickness for PtC deposits on Si–SiO_2 fabricated at 30 keV primary electron energy. The halo thickness is always found below 5% of the maximum deposit height (listed in legend) but below 3 nm for the investigated structures.

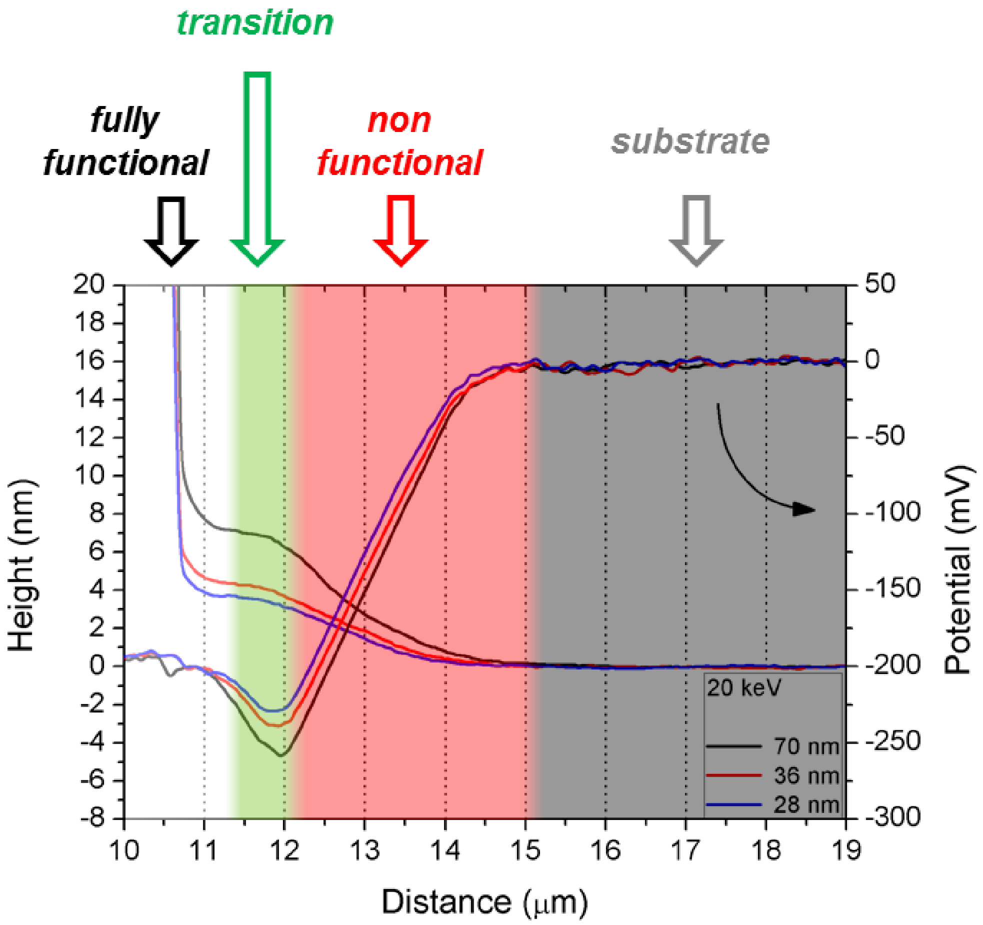

Figure S2: Correlated AFM height cross sections (left axis) together with corresponding surface potentials (right axis) for differently thick PtC deposits fabricated at 20 keV. The color code follows Figure 5 and indicates the plateaus aside the intended deposit partly as fully-funtional (white) followed by the transition area (green) which is partly conductive.

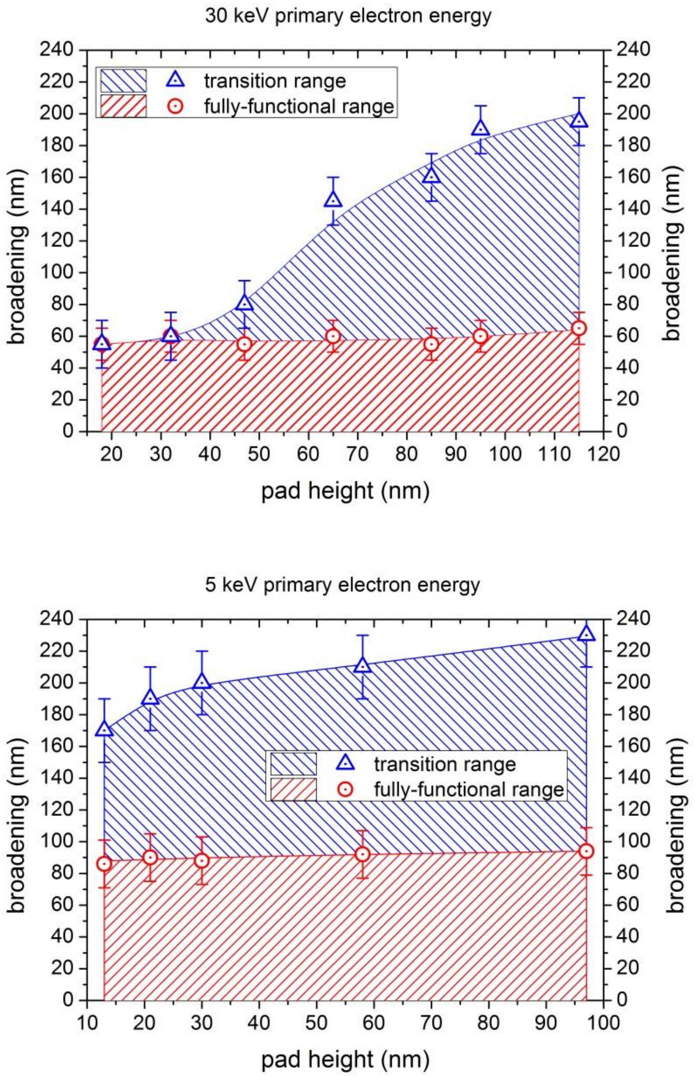

Figure S3: Considering the transition area as partly conductive and therefore influencing the lateral deposit sharpness with respect to its functionality, a scaling comparison for 30 keV (top) and 5 keV (bottom) is shown. As it can be seen the low energy deposits show slightly higher broadening even for thin deposits while highest energies and thinnest pads give highest lateral structure sharpness.

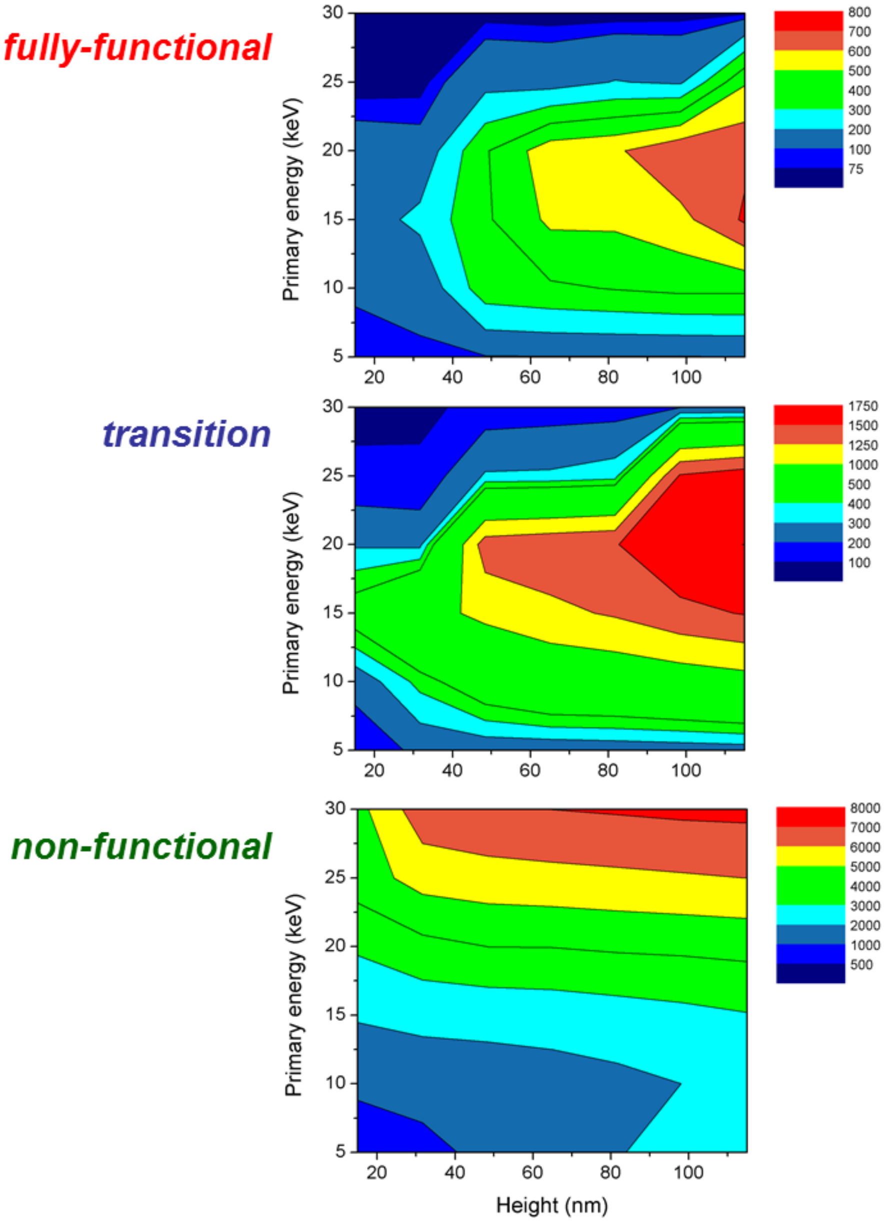

Figure S4: Contour plot representation of fully-functional (top), non-functional (bottom) and transition ranges with varying properties (center). As clearly evident intermediate energies should always be avoided while highest energies provide sharpest, functional edges.
